# A Five-Week-Old Twin With Profound Anemia: A Case Report of Asymmetric Congenital Babesiosis

**DOI:** 10.7759/cureus.22774

**Published:** 2022-03-02

**Authors:** Sulynn Walker, Elizabeth Coray, Julien Ginsberg-Peltz, Liza Smith

**Affiliations:** 1 Emergency Medicine, Baystate Medical Center, Springfield, USA; 2 Pediatric Emergency Medicine, Baystate Medical Center, Springfield, USA

**Keywords:** intraerythrocytic inclusions, thrombocytopenia, anemia, congenital infections, transplacental, babesiosis

## Abstract

We present a case report and review of the literature of congenital transmission of babesiosis in a five-week-old twin neonate evaluated for lethargy and difficulty with feeding. On presentation to the pediatric emergency department, the patient appeared pale and was found to be profoundly anemic with intraerythrocytic ringed parasites consistent with *Babesia microti *visible on a thin smear. The patient received a blood transfusion and was treated with a regimen of atovaquone and azithromycin with full recovery. The other twin remained asymptomatic with negative *Babesia* polymerase chain reaction testing.

## Introduction

Babesiosis is caused by intracellular parasites from the *Babesia* family, with* Babesia microti *being the predominant species in the United States. It is classically transmitted via the bite of the *Ixodes* *scapularis* (deer) tick. In 2019, the disease incidence in the United States was 0.86, with a total of 2,418 new cases [1]. A growing body of literature describes a steady increase in the incidence and distribution of *Ixodes* tick-vectored human disease [2-5]. Emergency providers need to be familiar with tick-borne diseases as they are predicted to continue to increase in prevalence, occur in geographic locations where they have not previously been reported, and occur in novel or unusual transmission patterns, as in our case, with increasing frequency [2-5]. There is little research available regarding the congenital transmission of tick-borne diseases, specifically babesiosis, and the available research tends to be in the form of case reports [6-14]. We present a case report of asymmetric transmission of babesiosis in a twin neonate presenting to the pediatric emergency department (ED) for lethargy and feeding difficulty. This is the first report addressing asymmetric congenital transmission in twins.

## Case presentation

A five-week-old female diamniotic dichorionic twin born at 36 5/7 weeks via cesarean section presented to the emergency department with pallor and increased lethargy. Over the previous day, the patient’s mother had noticed that she was more difficult to arouse and very pale compared to her twin brother. She was feeding with a similar frequency, however, with a decreased duration from 20 minutes to 10 minutes due to fatigue. Both twins were breastfed. The patient’s twin brother was asymptomatic. Prenatal history was pertinent for conception via in vitro fertilization. The patient’s mother had one febrile illness during pregnancy, occurring at approximately 23-24 weeks of gestation, which was associated with a maculopapular rash that resolved spontaneously. The patient’s mother made several trips to Cape Cod, Massachusetts, throughout the pregnancy.

**Figure 1:**
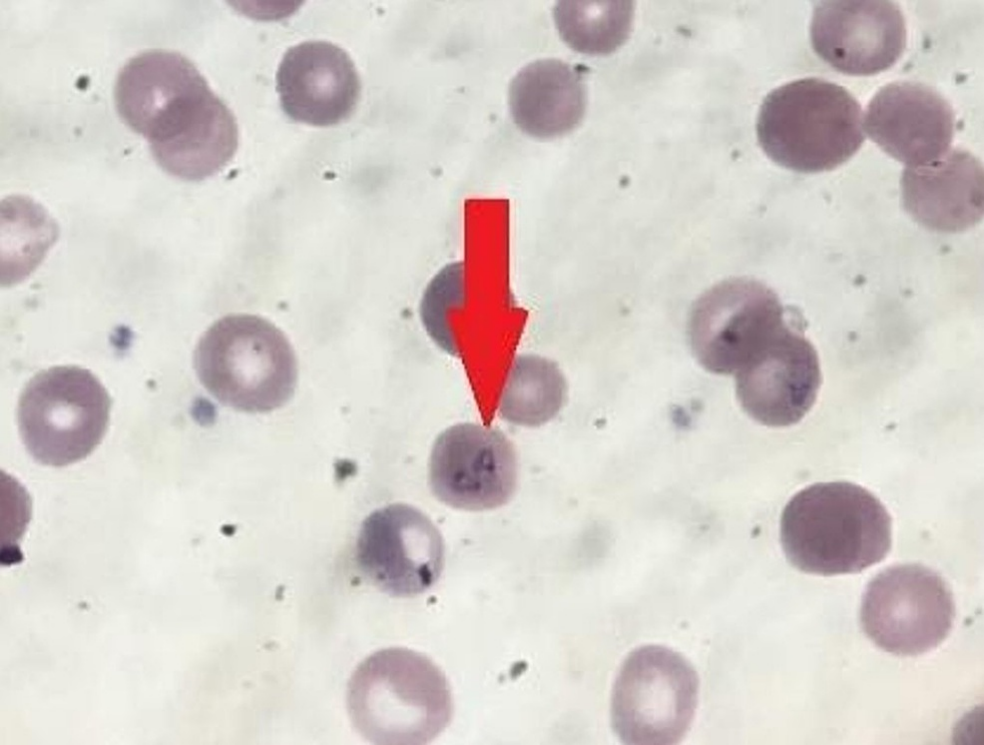
Blood smear with intraerythrocytic inclusions consistent with Babesia microti.

On examination, the patient was febrile to 100.4°F rectally, noticeably pale, but vigorous with mild tachypnea and tachycardia into the 170s-180s beats per minute. The abdomen was soft with the spleen palpable to 2 cm below the costal margin. A workup for neonatal sepsis was initiated, including complete blood count, blood cultures, urinalysis and urine culture, and lumbar puncture. The patient’s blood work was significant for a hemoglobin level of 3.4 gm/dL, with a hematocrit of 9.4%, leukocytosis to 16,000k/mm^3^, and platelets of 104k/mm^3^. Aminotransferases were elevated with aspartate aminotransferase (AST) of 186 U/L, alanine aminotransferase 116 U/L, and total bilirubin of 3.8 mg/dL. Because of the profound hematologic abnormalities, a routine thin smear was obtained which was significant for multiple intraerythrocytic ringed parasites consistent with *Babesia microti* (Figure 1). The extent of parasitemia was estimated at 2%, which is considered mild-to-moderate parasitemia, severe disease is >10%; however, disease classification may also be based on symptoms [15]. The patient received a blood transfusion with an initial bolus of 4 cc/kg of packed red blood cells and was started on intravenous atovaquone 20 mg/kg twice daily and azithromycin 10 mg/kg daily per the recommendation of the pediatric infectious disease team. The patient received multiple blood transfusions during the admission, and fluid status was closely monitored without any signs of fluid overload. Blood smears were repeated every 12 hours with clearance of parasitemia five days after presentation. The patient’s twin brother was also admitted for evaluation of *Babesia *infection and was found to be mildly anemic with a hemoglobin of 7.4 gm/dL; however, he was negative for *Babesia microti* by polymerase chain reaction (PCR) and negative for* Babesia* immunoglobulin (Ig)M. His IgG was positive, but it is unclear if this represents cleared infection or if this was Ig transferred transplacentally from maternal infection. The patient’s mother underwent testing as well, which returned *Babesia microti* IgG of 1:160 and IgM of <1:10, with a negative PCR consistent with cleared infection. On follow-up three months after the initial presentation, both twins were doing well and were completely asymptomatic.

## Discussion

*Babesia* species are intracellular protozoa that mediate disease by infecting and lysing red blood cells. Clinical manifestations can range from asymptomatic, self-limited disease to severe, fatal infections. Data derived from seroprevalence in endemic areas suggest that up to 40% of infections in children and 20% of infections in adults are asymptomatic [5]. Transmission of *Babesia microti* involves two hosts. First, an infected tick deposits eggs into a mouse [15,16]. An uninfected tick in the larval stage then takes a blood meal on the mouse and molts to the nymph stage. Then, a *Babesia*-infected tick in the nymph stage takes a blood meal on a human and infects the humans with the parasite, which then replicates in the erythrocyte [15,16]. The incubation can range from one week to months [15]. Common symptoms are non-specific and include fever, malaise, myalgias, headache, and nausea [15]. In a neonate, such as the patient presented in this case, it may be difficult to elucidate these symptoms. Hepatomegaly or splenomegaly may be appreciated on examination. Laboratory abnormalities are often suggestive of hemolysis and include anemia, elevated lactate dehydrogenase, low haptoglobin, and reticulocytosis [15,16]. Anemia was the first laboratory abnormality noticed in this patient on presentation and prompted the peripheral smear resulting in the diagnosis. One study reporting rates of neutropenia in babesiosis noted 60% of patients had an absolute neutrophil count less than 1,000/mm^3^ [17]. The mechanism of neutropenia in babesiosis is not known; however, in Malaria, another intracellular erythrocytic parasite, neutropenia is theorized to be related to the margination of leukocytes or to sequestration in the spleen [17]. Liver function tests, including alanine aminotransferase, aspartate aminotransferase, alkaline phosphatase, and total and indirect bilirubin, may be elevated as well [18]. In our patient, the anemia triggered a peripheral blood smear which revealed the erythrocytic inclusions. However, thin blood smear should be considered in any patient with fever of unknown origin who lives in or has traveled to an endemic area within two months [16]. PCR may also be useful in diagnosing babesiosis as well as assays for the detection of IgG and IgM. IgG and IgM may be useful for detection during the acute phase of illness [16]. In more severe cases, laboratory abnormalities tend to be more pronounced and are often associated with higher levels of parasitemia (generally >4%) [18]. Complications of babesiosis are typically associated with severe anemia and high parasitemia and include congestive heart failure, acute respiratory distress syndrome, disseminated intravascular coagulation, mental status changes, renal failure, and shock [19]. Risk factors for more severe disease include extremes of age, asplenia, and immunosuppression [18].

Some degree of anemia is expected in all infants because the erythrocytes containing fetal hemoglobin lyse and are replaced with new ones containing adult hemoglobin. The red blood cell nadir in healthy term infants is expected somewhere between six and twelve weeks, and typical hemoglobin levels are between 9.5 and 11 g/dL; however, values for preterm infants are often lower [20]. All of the previously reported cases of symptomatic infants diagnosed with suspected congenital babesiosis presented at six weeks of life or less, and their hemoglobin levels ranged from 6.3 to 11.6 g/dL [6-14]. While the value at the upper end of the range would be considered normal, it is occurring at a younger age than one would expect the nadir to occur. Our patient was more severely anemic than any previously reported case, with hemoglobin of 3.4 g/dL.

## Conclusions

Although case reports of congenitalbabesiosis exist, this is the first report describing asymmetric transplacental transmission in twins. Predictive models based on climate projections unanimously predict dramatic range expansions of *Ixodes* ticks and the tick-borne diseases they carry. The illnesses transmitted by the* Ixodes* tick are caused by various pathogens. As a result of this variation, there is no single model or classic disease transmission pattern. Tick-borne diseases, such as babesiosis, should be considered a part of the differential for anemia, thrombocytopenia, and neutropenia in an febrile infant as they are increasing in geographic range due to climate change. PCR blood testing, serologic testing, and blood smear examination are widely available, and clinicians noting thrombocytopenia, alone or in combination with anemia or neutropenia, in a febrile infant may want to consider sending these tests as part of their initial evaluation.
